# Long-term survival after anterior approach right hepatectomy combined with inferior vena cava thrombectomy using trans-diaphragmatic intrapericardial inferior vena cava occlusion: a case report and review of the literature

**DOI:** 10.1186/s12893-019-0568-7

**Published:** 2019-08-28

**Authors:** Yaodong Zhang, Zhengshan Wu, Ke Wang, Sheng Han, Changxian Li, Xiangcheng Li

**Affiliations:** Hepatobiliary Center, The First Affiliated Hospital of Nanjing Medical University; Key Laboratory of Liver Transplantation, Chinese Academy of Medical Sciences; NHC Key Laboratory of Living Donor Liver Transplantation, Nanjing, China

**Keywords:** Hepatocellular carcinoma, Inferior vena cava, Tumor thrombosis

## Abstract

**Background:**

Presence of inferior vena cava tumor thrombosis (IVCTT) is an unfavorable factor to prognosis for patients with hepatocellular carcinoma (HCC).

**Case presentation:**

Herein we report a case of HCC with IVC tumor thrombosis extending from the right hepatic vein (RHV) to the IVC, but it had not infiltrated the right atrium. Anterior approach right hepatectomy combined with IVC thrombectomy using trans-diaphragmatic IVC occlusion was performed for this patient. The patient is alive with disease-free at 32 months after treatment. A literature review was also performed. This case was demonstrated with the details and concepts of surgery.

**Conclusion:**

This case suggested that surgical resection of HCC involving the IVC, but still outside the right atrium (RA), could offer satisfactory surgical outcomes in selected patients.

## Background

Primary hepatocellular carcinoma (HCC) accounts for 90% of primary liver cancer in China [1]. The presence of inferior vena cava tumor thrombosis (IVCTT) in patients with HCC is considered critically closely associated with poor prognosis [2–6]. The staging system issues by the Barcelona Clinic Liver Cancer (BCLC) / American Association for the Study of Liver Diseases (AASLD) recommends the palliative care for patients in whom unsatisfactory survival is predicted [7]. With the advances in surgical techniques and increases in the quality of perioperative care, surgical resection is associated with acceptable prognosis in HCC patients with IVCTT, especially patients who undergo R0 resection [8–10].

Herein, we report an HCC patient with IVCTT who underwent hepatectomy combined with IVC thrombectomy via the anterior approach and we summarized the treatments and outcomes of previous published studies that covered similar patients.

## Case presentation

A 49-year-old male Chinese HCC patient with chronic hepatitis B virus (HBV) infection (having lasted more than 20 years) and cirrhosis. The laboratory results showed the following: alanine aminotransferase (ALT) 43.4 U/L, aspartate aminotransferase (AST) 35.7 U/L, alkaline phosphatase (ALP) 200.5 U/L, gamma-glutamyl transpeptidase (GGT) 188.1 U/L, bilirubin (TBil) 15.5 μmol/L, and a-fetoprotein (AFP) > 2000 μg/L. Liver function of the patient was Child-Pugh A grade and preoperative indocyanine green retention rate (ICG) was 10.5%.

**Fig. 1:**
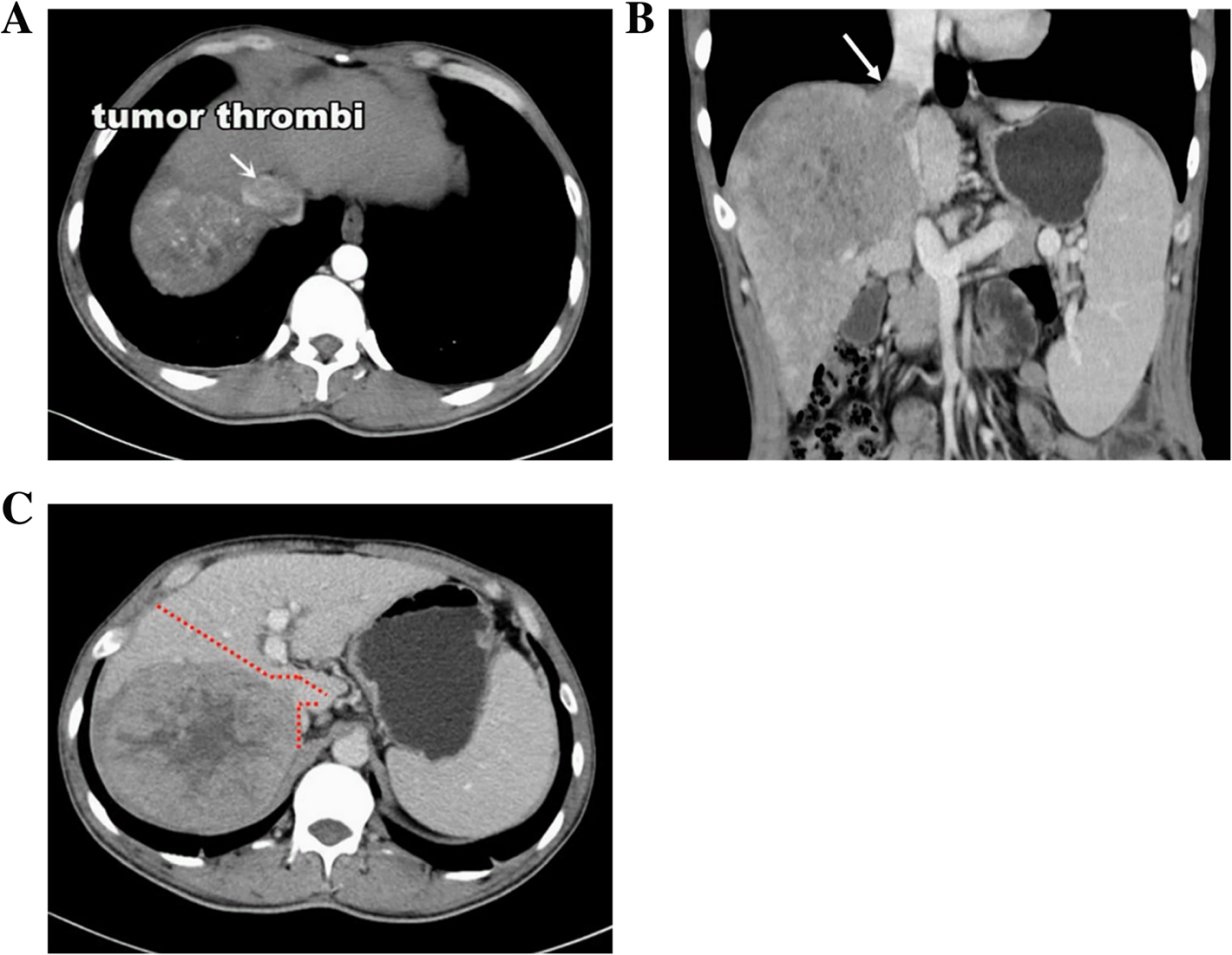
**a** CT scan showing a huge HCC located in the right hepatic lobe with tumor thrombus (arrow) entering the IVC. **b** Coronal CT suggests IVTT had passed the diaphragm level, but had not enter the right atrium (arrow). **c** Anterior approach right hepatectomy combined with IVCTT thrombectomy

Computed tomography (CT) showed a large mass about 11.3 × 9.9 cm^2^ in size in the right hepatic lobe, and a tumor thrombus (TT) in the right hepatic vein (RHV) extending into the IVC (Fig. 1a and b).

Using both his medical history and imaging findings, he was diagnosed with HCC associated with IVCTT. The clinical stage was BCLC stage C. The patient had no symptoms of right heart failure or pulmonary embolization at admission. Considering that the patient had normal hepatic function without distant metastasis, anterior approach right hepatectomy combined with IVC thrombectomy using trans-diaphragmatic intrapericardial IVC occlusion was planned for this patient (Fig. 1c).

**Fig. 2:**
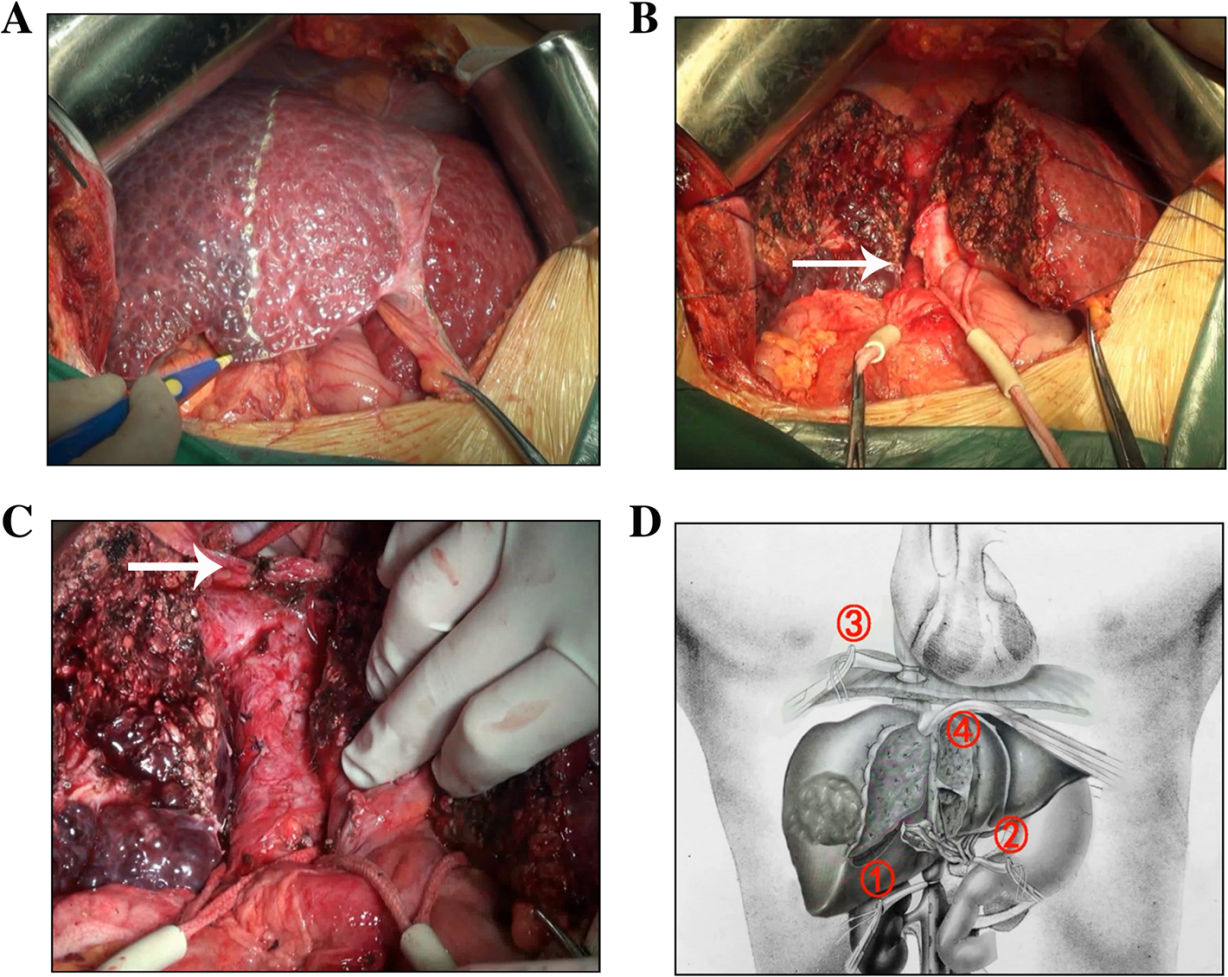
**a** Ligation of the right hepatic artery and portal vein. **b** The suprarenal IVC and portal vein were dissected and encircled (arrow). **c** The supradiaphragmatic IVC was encircled through a vertical incision of the diaphragm (arrow). **d** The sequence of total hepatic vascular exclusion: ① Suprarenal IVC ② The portal vein ③ Supradiaphragmatic IVC ④ Retrohepatic IVC

Surgery was performed via a subcostal inverse-L-shaped incision. At laparotomy, a tumor located in the right lobe of cirrhotic liver and no detectable ascites or peritoneal metastasis was observed. After the right hepatic artery and the right portal vein branch were ligated, hepatic parenchymal resection was performed using the clamp-crushing technique with inflow occlusion (Pringle’s maneuver) following the demarcation (Fig. 2a). Then the suprarenal IVC and portal vein were dissected and taped from the caudate lobe (Fig. 2b). The retrohepatic IVC below the confluence of the common channel of the left and middle hepatic veins was encircled by a vascular clamp. The diaphragm was transected via a vertical incision exposing the right atrial appendage. Then intraoperative ultrasonography was used to show that a TT in the RHV, involving the IVC, but it had not entered in the right atrium. The supradiaphragmatic IVC was encircled though trans-diaphragmatic intrapericardial IVC (Fig. 2c). The sequence of total hepatic vascular exclusion is shown in Fig. 2d and the IVTT was then removed en bloc successfully with Babcock forceps, the whole removal of IVCTT with IVC exclusion cost 20 min. The total operation required 481 min and the intraoperative hemorrhage was 900 ml.

**Fig. 3:**
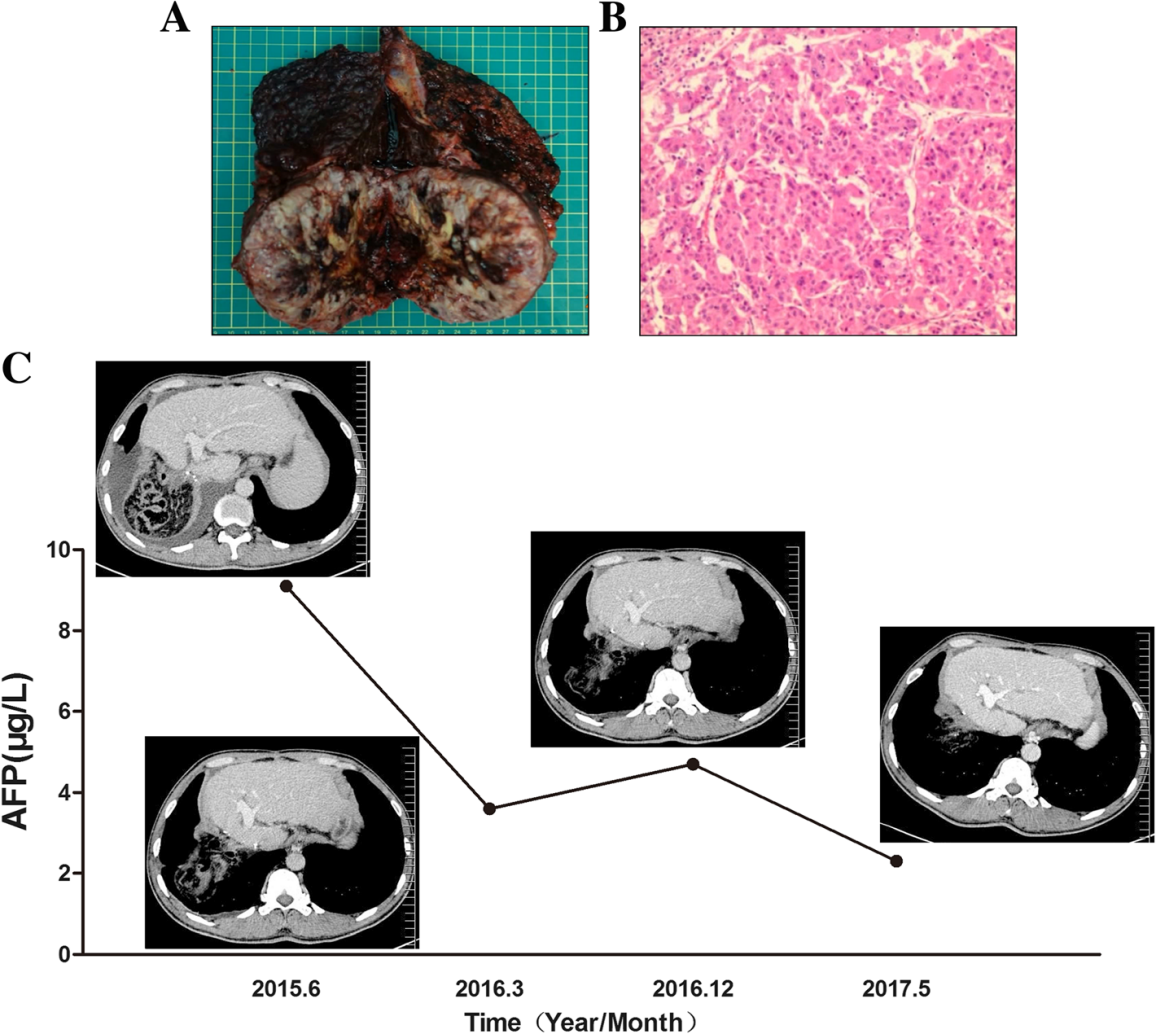
**a** The resected tumor specimens. **b** Hepatocellular carcinoma, II-III, giant size, size 10 × 11 × 13 cm, visible intravascular tumor thrombus, incision without tumor residual (HE staining, × 400). **c** CT examinations and laboratory results of AFP showed no signs of recurrence or metastasis 32 months after the surgery

The macroscopic findings of tumor measured 10 × 11 × 13 cm^3^ and the TT measured 3.0 × 2.0 cm2 (Fig. 3a). Postoperative histological diagnosis showed moderately differentiated HCC (grade II-III Edmondson) had invaded the right hepatic vein with hepatic fibrosis and intravascular tumor thrombus. No positive resection margins or local lymph node metastasis were observed microscopically (Fig. 3b). The TNM stage was T3bN0M0.

Postoperative recovery was uneventful. The patient was discharged with few adverse events after the operation. The patient was disease-free at 32 months after the initial treatment (Fig. 3c).

## Discussion and conclusions

Despite advances in perioperative therapy and multidisciplinary therapy, IVCTT-presenting HCC is still a significant factor for a dismal prognosis of HCC patients [3, 11, 12]. The median survival time after surgical resection for HCC with IVCTT was significantly better than that without surgical resection or in patients treated with sorafenib (47.4 vs 4/10.7 months) [3, 7]. Regarding prognosis, we reviewed 33 case reports of HCC with IVCTT (Table 1) and found the mean patient age to be 55.8 ± 15.4 years, and the mean time that elapsed between diagnosis and treatment was 20 ± 22.8 months in the 17 patients who underwent liver resection and TT, the mean time that elapsed between diagnosis and treatment was 9 ± 4.5 months in the 7 patients who underwent resection of TT alone, and the mean time that elapsed between diagnosis and treatment was 10.4 ± 12.3 months in the 12 patients who received other oncology therapy. These data indicated that aggressive radical resection of thrombus combined with hepatectomy or tumor thrombus extraction alone, might yield better survival than other non-surgical treatment.

**Table 1:** Reported cases of hepatocellular carcinoma with IVC involvement

No	Literature	Age/Sex	Location	Size	IVC Involvement	Treatment Technique	Additional Treatment	Survival	Outcome
1	Tastekin [13]	24/male	Rt lobe	6 × 6 × 4 cm	III LV	Resection of liver tumor	Resection of tumor thrombus	18 mo	Dead
2	Ehrich [14]	62/male	Lt lobe	ND	III RA	Resection of tumor thrombus	Radiation therapy	12 mo	Alive
3	Li [15]	66/male	Rt lobe	12 cm	III RA	Resection of liver tumor and tumor thrombus	No	6 mo	Dead
4	Oncale [16]	76/male	Rt lobe	12 × 8 cm	II	Sorafenib	No	14 mo	Dead
5	Miller [17]	30/male	Extensive involvement	ND	III RA	Resection of tumor thrombus	Radiation therapy and immunotherapy	6 mo	Alive
6	Chang [4]	57/male	RAS	9.6 × 8.6 cm	III RA	Thalidomide	No	12 mo	Alive
		76/male	MHV	2 cm	III RA	TACE	Thalidomide	18 mo	Dead
		67/female	Seg5, Seg8	7 cm	III RA	Thalidomide	No	5 mo	Alive
7	Inoue [39]	67/male	Seg2, Seg3	1.2 cm	III RA	Resection of liver tumor and tumor thrombus	No	27 mo	Alive
8	Luo [18]	35/male	Lt lobe, RAS	ND	III RA	Percutaneous microwave ablation	No	6 mo	Dead
9	Goto [19]	36/female	Rt lobe	ND	III RA	Resection of tumor thrombus	TACE	7 mo	Dead
10	Kawakam [20]	66/female	Extensive involvement	ND	III RA	Symptomatic treatment	TACE	5 mo	Dead
11	Sun [21]	45/male	Lt lobe	ND	III RA	TACE	No	46 mo	Alive
12	Georgen [22]	22/female	Seg7	6 cm	III RA	Resection of liver tumor and tumor thrombus	No	30 mo	Dead
13	Noguchi [23]	66/male	Rt lobe	2 cm	III RA	Symptomatic treatment	No	1 mo	Dead
14	Ohta [43]	60/male	Rt lobe	ND	III RA	Resection of liver tumor and tumor thrombus	Sorafenib	10 mo	Alive
15	Sengodan [24]	44/male	Rt lobe	5.6 × 7.6 × 5.5 cm	III RA	Sorafenib	Anticoagulation	2 mo	Dead
16	Hayashida [25]	72/male	Lt lobe	ND	III RA	Resection of tumor thrombus	TACE	6 mo	Dead
17	Sawada [26]	62/male	Extensive involvement	ND	III RA	Glypican-3-derived peptide vaccination	TACE, sorafenib	56 d	Dead
18	Shivathirthan [40]	71/male	Seg 7	ND	III RA	Resection of liver tumor and tumor thrombus	TACE	ND	ND
19	Miyazawa [27]	55/male	RAS	7 cm	III RA	Resection of liver tumor and tumor thrombus	No	12 mo	Alive
20	Li [28]	64/male	Seg 6, Seg7	5.3 × 3.6 cm	III RA	Resection of liver tumor and tumor thrombus	No	6 mo	Dead
21	Wu [42]	42/male	RAS	1.5 cm	III RA	Resection of liver tumor and tumor thrombus	TACE	14 mo	Dead
22	Giuliani [29]	47/male	Lt lobe	Mutiple	III RA	External beam radiation therapy	No	7 mo	Dead
23	Leo [30]	45/male	Lt lobe	ND	III RA	Resection of liver tumor and tumor thrombus	No	6 mo	Alive
24	Florman [3]	55/male	Lt lobe	18 × 3 × 7 cm	III RA	Resection of liver tumor and tumor thrombus	No	3 mo	Alive
25	Sung [31]	71/male	Rt lobe	ND	III RA	Resection of tumor thrombus	Sorafenib	7 mo	Alive
26	Ohwada [32]	77/female	LLS	10 cm	III RA	Resection of liver tumor and tumor thrombus	No	3 mo	Alive
27	Lin [33]	57/male	Rt lobe	4.5 cm	III RA	Resection of liver tumor and tumor thrombus	No	3 D	Dead
28	Yogita [34]	61/male	LMS	3 cm	III RA	Resection of liver tumor and tumor thrombus	No	56 mo	Dead
29	Masaki [35]	47/male	Seg 8	3 × 2.5 × 2 cm	III RA	Resection of tumor thrombus	TAE and radiation therapy	8 mo	Dead
		48/male	Seg 7	ND	III LP	Resection of tumor thrombus in the IVC and LPA	TAE	29 D	Dead
30	Kashima [20]	66/male	Rt lobe	10 × 7 cm	III RA	Resection of liver tumor and tumor thrombus	TACE	59 mo	Alive
31	Fujisaki [36]	38/female	LLS	8 × 8 cm	III RA	Resection of liver tumor and tumor thrombus	No	15 mo	Alive
32	Dazai [37]	42/male	Rt lobe	ND	III RA	TACE	No	7 mo	Dead
33	Kurahashi [38]	81/male	Rt lobe	11.5 cm	III RA	Resection of liver tumor and tumor thrombus	Oncologic treatment	72 mo	Alive

**Fig. 4:**
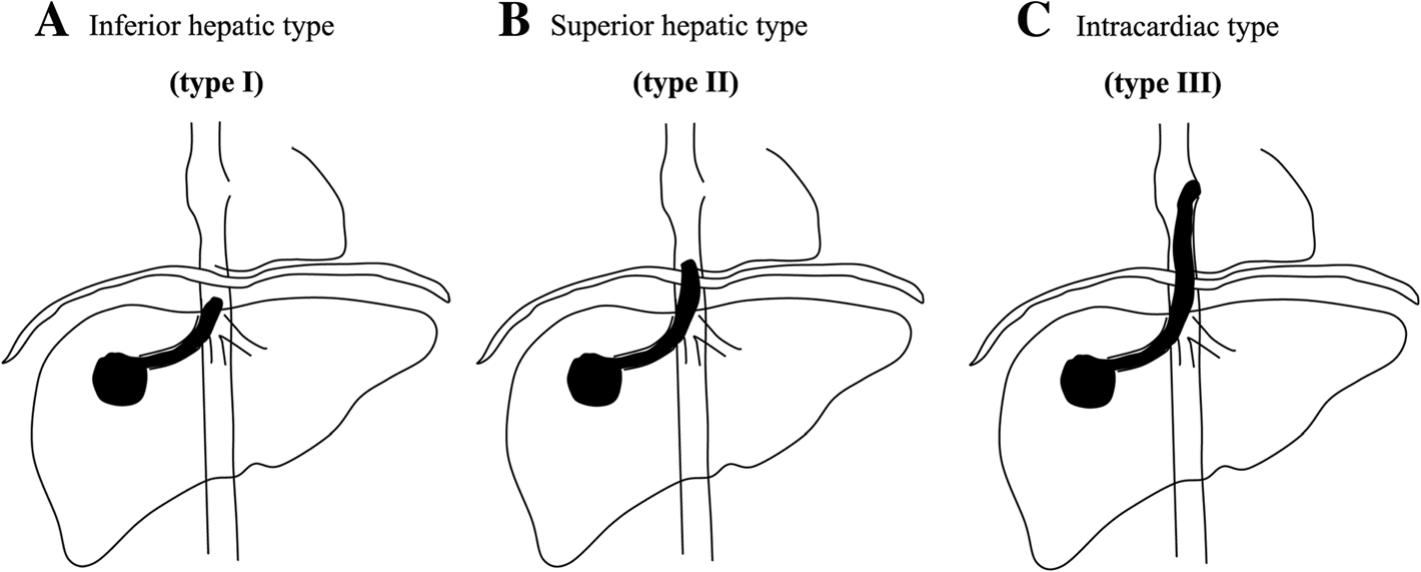
IVCTT-presenting HCC is classified into three types. **a** The TT located within the subdiaphragmatic IVC; **b** The IVCTT extended above the diaphragm, but it had not infiltrated the RA; and (**c**) The TT extended over the diaphragm and had advanced into the RA

*IVC* inferior vena cava, *LLS* left lateral segment, *LMS* left medial segment, *Lt* left, *MHV* middle hepatic vein, *ND* not described, *RA* right atrium, *RAS* right anterior segment, *Rt* right, *Seg* segment, *TACE* transcatheter arterial chemoembolization, *TAE* transcatheter arterial embolization, *LPA* Left pulmonary artery, *LP* left pulmonary, *mo* months, *II* type II (mentioned in Fig. 4), *III* type III (mentioned in Fig. 4)

A previous clinical study defined the subtypes of IVCTT-presenting HCC into three types based on the anatomic locations of the IVCTT and heart. The TT located within the subdiaphragmatic IVC was defined as the inferior hepatic type (Type I) (Fig. 4a), and the IVCTT extended above the diaphragm, but it had not infiltrated the RA. It was defined as superior hepatic type (Type II) (Fig. 4b), for the intracardiac type (Type III). The TT extended over the diaphragm and had entered the RA (Fig. 4c) [12].

With the advances in surgical techniques, increasing numbers of successful simultaneous resection of HCC with IVC tumor thrombosis have been reported [39]. Kokudo et al. reported that the 1-year and 3-year survival rates were 81 and 32%, respectively, and the median survival time was 16.7 months [8]. Wang et al. reported that the 1-year, 3-year, and 5-year survival rates were 68, 22.5, and 13.5% and the median survival time was 19 months [10]. These data indicated that resection of HCC and IVCTT might result in better survival than other non-surgical therapies.

Cardiopulmonary bypasses (CPB) and total hepatic vascular exclusion (THVE) have been reported in most previous resection cases [40–43]. However, these technologies have reportedly been associated with the risk of massive hemorrhage and severe vascular injury. Although other new technologies, such as venous bypass, total circulatory arrest with exsanguinations, and concomitant hypothermia, can reduce these risks, technical complexity is still the main limitation of these procedures [42, 44]. The basis of our technique is that the IVCTT undergo no or minimal adhesion to the venous wall on any macroscopic scale. Similar ideas have been mentioned in two previous articles. We have simplified the surgery procedure and difficulty based on the new surgical concepts. It has been suggested that this surgical procedure could be suitable for HCC patients with IVCTT extended above the diaphragm without entering the RA, and it could reduce the risk of bleeding and vascular injury to a considerable intent.

No-touch surgery is difficult to perform given the anatomical characteristics of the liver [45]. Recent studies have confirmed that tumor cells diffuse more easily through the portal vein or hepatic vein during the conventional hepatectomy than through other veins and resulting in early recurrence [46, 47]. Recently, some studies have reported that anterior approach hepatectomy, because involves less manipulation of the liver, can reduce the rate of recurrence of postoperative liver cancer, and extend patient survival [48]. Clinical, randomized controlled studies reported that half-hepatectomy in the anterior approach significantly reduced the risk of blood loss, improved the survival rate, ensured surgical vision, and reduced the perioperative mortality rate [49].

During this procedure, a process termed the peeling-off technique was performed because tumor thromboses do not adhere to the wall of the IVC. Previous studies have proved that the peeling off technique can improve the surgical outcome of HCC with portal vein tumor thrombus [50, 51]. We reported the resection for HCC with IVCTT using the peeling off technique and the long-term survival outcomes are comparable to the current case reports. The low incidence of IVC tumor thrombus in HCC should not preclude the development of new surgical approaches because the peeling-off technique is a minimally invasive approach and deserves further investigation.

In this case, because the IVCTT is above the diaphragm but still outside the RA, the intrathoracic IVC is approached by an abdominal incision of the diaphragm, without the need for a median sternotomy or thoracotomy [52, 53]. This method prevents any need for splitting the sternum and also reduced surgical trauma, keeping the IVC hiatus and preserved the normal anatomical structures, and offered good surgical exposure.

In conclusion, radical resection of both HCC and IVCTT could be a practical surgical option and useful therapeutic modality for achieving long-term survival or HCC patients with IVCTT extending to the IVC, especially for those IVCTT extending over the diaphragm but outside the RA.

## Data Availability

All data generated or analyzed during this study are included in this published article.

## Abbreviations

CT: Computed tomography
HBV: Hepatitis B virus
HCC: Hepatocellular carcinoma
IVC: Inferior vena cava
TACE: Transcatheter arterial chemoembolization
TAE: Transcatheter arterial embolization
TT: Tumor thrombosis
